# Altered Serum Immunological and Biochemical Parameters and Microbiota Composition in Patients With AN During Realimentation

**DOI:** 10.3389/fnut.2021.680870

**Published:** 2021-08-02

**Authors:** Radka Roubalova, Petra Prochazkova, Jiri Dvorak, Martin Hill, Hana Papezova, Jakub Kreisinger, Josef Bulant, Alena Lambertova, Petra Holanova, Martin Bilej, Helena Tlaskalova-Hogenova

**Affiliations:** ^1^Laboratory of Cellular and Molecular Immunology, Institute of Microbiology of the Czech Academy of Sciences, Prague, Czechia; ^2^Department of Steroids and Proteohormones, Institute of Endocrinology, Prague, Czechia; ^3^Department of Psychiatry, First Faculty of Medicine, Charles University and General University Hospital in Prague, Prague, Czechia; ^4^Department of Zoology, Faculty of Science, Charles University, Prague, Czechia; ^5^Department of Paediatrics and Inherited Metabolic Disorders, First Faculty of Medicine, Charles University and General University Hospital in Prague, Prague, Czechia

**Keywords:** anorexia nervosa, realimentation, alpha-melanocyte stimulating hormone, immune system, autoantibody, *Enterobacteriaceae*, microbiota

## Abstract

Anorexia nervosa (AN) is a life-threatening psychiatric disorder with not well-described pathogenesis. Besides the genetic and sociological factors, autoimmunity is also considered to take part in AN pathogenesis. We evaluated general serological factors showing the physiological state of 59 patients with AN at hospital admission and their discharge. We detected the altered levels of some general biochemical and immunological parameters. We also detected decreased levels of appetite-regulating alpha-melanocyte stimulating hormone (α-MSH) in patients at hospital admission. Moreover, elevated anti-α-MSH IgM levels and decreased anti-α-MSH IgA levels were observed in patients with AN. Therefore, we analyzed the gut microbiota composition with special focus on α-MSH antigen-mimetic containing microbes from the *Enterobacteriaceae* family. We correlated gut bacterial composition with anti-α-MSH Ig levels and detected decreasing IgG levels with increasing alpha diversity. The upregulation of pro-inflammatory cytokines IL-6, IL-17, and TNF-α were detected in patients with AN both prior and after hospitalization. We also evaluated the treatment outcome and improvement was observed in the majority of patients with AN. We provide new data about various serum biochemical parameters and their changes during the patients' hospitalization, with emphasis on the immune system, and its possible participation in AN pathogenesis.

## Introduction

Anorexia nervosa (AN) is a disease characterized by extremely reduced body weight resulting from food intake reduction, substantial fear of weight gain, and distorted self-image projection (1). This psychiatric disorder is associated with great mortality rates (2). Genetic factors influence the risk of AN development, psychological and sociocultural influences may trigger disease onset, and a variety of other factors can sustain the illness (1). The gut microbiome is involved in human energy homeostasis and also in AN development (3–6). AN is associated with many psychiatric comorbidities, such as depression, anxiety, and obsessive-compulsive disorder (1). Besides, patients with AN display a broad variety of health problems affecting almost every body system due to malnutrition. Gastrointestinal disorders such as slowed gastric emptying, superior mesenteric artery syndrome, and duodenogastric reflux were described in patients with AN (7, 8). Further, various cardiac, pulmonary, hematological, and musculoskeletal disorders are connected with this disease (9). In patients with AN, changes in multiple endocrine axes were observed. These changes are mostly adaptive to reduced energy intake, however, they can be connected to various deleterious consequences, such as decreased bone marrow mineral density and negative impact on mood, emotions, and cognition (10). Appetite-regulation disruption influences the aberrant feeding behavior in eating disorders (11). In patients with AN, increased orexigenic ghrelin levels, and conversely, decreased levels of anorexigenic leptin and alpha-melanocyte stimulating hormone (α-MSH) were described (11–13). Interestingly, autoantibodies directed against these peptides detected in patients' serum are supposed to participate in AN pathophysiology (14–16). Caseinolytic protease B (ClpB), α-MSH conformational mimetic produced by the *Enterobacteriaceae* family induces the production of antibodies cross-reacting with human α-MSH (17). Further, IgG from patients with AN can form immunocomplexes with α-MSH, which enhances the neuropeptide action on melanocortin 4 receptor [MC4R; (18)], which is involved in feeding behavior.

Immune system participation in some psychiatric disorders pathogenesis is being widely considered (19). In patients with AN, the immune system is impaired, nevertheless, the distortion is not as serious as in the case of malnourished individuals (20). This is probably due to the relatively preserved protein and vitamin intake of patients with AN compared to typical malnutrition. This may explain why patients with AN do not have an increased infection risk despite their undernourished state, while malnourished people often exert an elevated infection risk (21).

The objectives of the present work were to evaluate the specific anthropometric and physiological parameter changes during AN inpatients' realimentation, and to assess their participation in AN pathophysiology and progression. Besides, the potential effect of microbiota disruption on appetite regulation was assessed. Levels of α-MSH and anti-α-MSH antibodies were determined and association between these antibodies and microbial alpha diversity was shown. We hypothesize that these parameter values and their changes during hospitalization are interconnected with AN severity and duration. The advantage of our study is its longitudinality and large cohort of analyzed patients with AN and healthy controls.

## Materials and Methods

### Participants

Forty-five restrictive and fourteen purgative AN women inpatients [age 23 (19, 27); BMI 14.4 (13.4, 15.9)] admitted to the Centre for Eating Disorders of the 1^st^ Faculty of Medicine of Charles University and of the General University Hospital in Prague were recruited to the present study. All patients were evaluated by two eating disorders-specialized medical doctors to meet criteria of DSM-V diagnoses for AN (22) and M.I.N.I to exclude comorbidity cases. The EDE-Q questionnaire was used to differentiate eating disorder subtype (23). Only clinically stable patients with AN without psychosis or current psychoactive substances abuse were included. The study was conducted in accordance with the Declaration of Helsinki, and the protocol was approved by the Ethics Committee of General University Hospital in Prague (16/16; approval date 21/4/2016). Seven of these patients terminated the therapy prematurely, but we included their initial samples into the analyses. Sixty-seven healthy women of age 24 (22, 28.5) and body mass index (BMI) 21.9 (19.9, 23.7) were screened for potential eating disorder by SCOFF Questionnaire, and were recruited from university students, office workers, and university employees. Exclusion criteria for all participants were pregnancy or breastfeeding and active infection. Healthy volunteers did not have a history of eating disorders or any other psychiatric disease. All participants signed an informed consent form.

Anthropometric measurements of patients with AN and healthy controls (height, BMI, body fat, waist and hip circumference; Table 1) were performed by nursing staff. Blood samples were collected from the cubital vein of all participants early in the morning. All participants were fasting for 12 h before a blood collection. Simultaneously, participants provided a stool sample, which was immediately frozen at −80°C. Patients with AN were asked for blood and stool samples at the beginning (AN1) and at the end (AN2) of their hospitalization. The blood was collected on the second day of the hospitalization, the stool was collected at the first defecation after hospital admission. Second blood and stool collection was performed on the last day of their hospitalization. Two days prior to stool collection, participants were asked not to drink alcohol, coffee, black tea; not to eat chocolate, products containing cacao, bananas, nuts; and not to take probiotics or aspirin. The treatment outcome of patients with AN at their discharge was evaluated (Supplementary Table 1).

**Table 1:** Comparison of anthropometric parameter values in healthy controls and patients with AN at the beginning (AN1) and at the end (AN2) of hospitalization.

**Variable**	**Control**	**AN1**	**AN2**	**Δ**	***P*-value for Δ**	***P*** **-value**			***P*-value for Kruskal-Wallis test**
						**AN1 vs. AN2**	**AN1 vs. C**	**AN2 vs. C**	
Age (years)	24 (22, 28.5)	23 (19, 27)							
Hospitalization (days)			51 (38.5, 64)						
Disease duration (months)		60 (36, 126)							
Height (cm)	169 (167, 173)	165 (162, 170)							
BMI (kg/m^2^)	21.9 (19.9, 23.7)	14.4 (13.4, 15.9)	17.1 (15.5, 18.1)	2.18 (1.53, 3.2)	<0.001	^***^	^***^	^***^	<0.001
Body fat (%)	24.2 (21.1, 28.7)	3 (3, 7.2)	9 (3.43, 15.2)	3.4 (0, 7)	<0.001	^*^	^***^	^***^	<0.001
Waist (cm)	70 (66, 74)	57.5 (55, 61)	64 (61, 66.8)	6 (4, 8)	<0.001	^***^	^***^	^***^	<0.001
Hip (cm)	94 (90.5, 100)	77 (74, 80)	83 (79.3, 84.8)	4 (3, 7)	<0.001	^**^	^***^	^***^	<0.001

*Changes during hospitalization (calculated as the values at hospital admission and values at hospital discharge) were evaluated by Wilcoxon's paired test corrected for ties. The multiple comparisons between groups were evaluated by the Kruskal-Wallis Z test followed Dunn's multiple comparisons with Bonferonni correction. Both Kruskal-Wallis test and Wilcoxon's test were corrected for multiplicity using Bonferroni correction. The results are shown as median with quartiles. AN1—patients with anorexia nervosa before treatment, AN2—patients with anorexia nervosa after treatment; Δ represents the absolute change calculated as the value after intervention - basal value; nControl = 67, nAN1 = 52, nAN2 = 52;*

* *p < 0.05,*

** *p < 0.01,*

*** *p < 0.001. NS, non-significant*.

### Biochemical Analysis of Blood Samples

Blood samples from patients with AN at hospital admission (AN1), at their discharge (AN2), and from healthy controls were used to determine the levels of total protein, albumin, globulins (alpha 1-, alpha 2-, beta-, gamma-), immunoglobulins (IgG, IgA, IgM, IgE), C-reactive protein (CRP), cholinesterase (CHE), triacylglycerols (TAG), thyrotropin (TSH), and thyroxine (fT4) (Table 2; Agilab, Czech Republic). Normal adult women reference ranges are shown in Table 2. The comparison of individual parameter levels in healthy controls, AN1, and AN2 was analyzed by the Kruskal-Wallis test with Bonferroni correction. The comparison of changes during hospitalization was evaluated by Wilcoxon paired test corrected for ties. Liver parameters [bilirubin, alanine transaminase (ALT), aspartate transaminase (AST), γ-glutamyltransferase (GGT), alkaline phosphatase (ALP)], pancreatic parameters [total serum amylase (AMS), pancreatic amylase (AMS-P)], cholesterol, renal parameters (urea, creatinine, uric acid), and ions (Na, K, Cl, Ca, P, Mg) were assessed only in AN1 patients (Supplementary Table 2).

**Table 2:** Comparison of serum parameter values in healthy controls and patients with AN at hospital admission (AN1) and discharge (AN2).

**Variable**	**Control**	**AN1**	**AN2**	**Δ**	***P*-value for Δ**	***P*** **-value**			***P*-value for Kruskal-Wallis test**	**Normal adult women reference ranges**
						**AN1 vs. AN2**	**AN1 vs. C**	**AN2 vs. C**		
Total protein (g/l)	69.2 (66.7, 71.5)	66.7 (63.6, 70.5)	67.6 (64.6, 69.9)	0.2 (−3.65, 3.23)	NS		^**^	^*^	<0.05	65–85
Albumin (%)	57.5 (55.4, 59.8)	60.2 (58.7, 62.8)	58.5 (57, 60.3)	−2.4 (−4.3, −0.675)	<0.001	^**^	^***^		<0.001	55–69
Albumin (g/l)	40.4 (38.2, 41.7)	40.5 (38.1, 42.2)	39.2 (38.1, 40.5)	−1.55 (−3.08, 0.325)	NS				NS	-
Alpha 1 globulin (%)	2.5 (2.2, 2.8)	2.2 (1.9, 2.5)	2.2 (2, 2.7)	0 (−0.2, 0.2)	NS		^**^		NS	1.5–4
Alpha 1 globulin (g/l)	1.7 (1.55, 1.9)	1.5 (1.3, 1.7)	1.5 (1.35, 1.8)	0 (−0.2, 0.2)	NS		^***^	^**^	<0.01	-
Alpha 2 globulin (%)	11.9 (10.9, 12.9)	11.9 (10.9, 12.3)	12.4 (11.9, 13.3)	0.8 (0.5, 1.63)	<0.001	^**^		^*^	NS	8–12
Alpha 2 globulin (g/l)	8.1 (7.45, 8.95)	7.8 (7.05, 8.4)	8.4 (7.8, 9)	0.6 (0.2, 1.4)	<0.001	^**^			NS	-
Beta globulin (%)	11.9 (11, 13)	11 (10.2, 11.9)	12.4 (11.3, 13)	1.4 (0.975, 2.13)	<0.001	^***^	^***^		<0.001	7–15
Beta globulin (g/l)	8.4 (7.7, 9.1)	7.3 (6.6, 8.1)	8.3 (7.6, 9)	0.8 (0.3, 1.9)	<0.001	^***^	^***^		<0.001	-
Gamma globulin (%)	16 (14.2, 17.7)	14.1 (12.2, 16.2)	14 (12.6, 16.3)	0 (−0.95, 0.925)	NS		^**^	^**^	<0.05	9–18
Gamma globulin (g/l)	11.1 (9.65, 12.7)	9.6 (7.7, 11.4)	9.7 (8.15, 11.1)	0.2 (−0.625, 0.875)	NS		^***^	^**^	<0.01	-
CRP (mg/l)	0.76 (0.34, 1.7)	0.61 (0.36, 0.89)	0.56 (0.18, 1.04)	0.0219 (−0.398, 0.511)	NS				NS	2–8
Cholinesterase (?kat/l)	108 (100, 123)	93 (77.5, 111)	104 (95.5, 118)	14 (4, 23.5)	<0.001	^*^	^***^		<0.01	85.2–195.4
TAG (mmol/l)	0.85 (0.59, 1.08)	0.85 (0.59, 1.02)	0.92 (0.68, 1.2)	0.07 (−0.06, 0.333)	NS				NS	0.45–1.7
TSH (mIU/L)	2.36 (1.92, 3.44)	2.11 (1.43, 2.7)	2.3 (1.78, 3.41)	0.3 (−0.423, 0.749)	NS				NS	0.3–3.5
fT4 (pmol/l)	14.8 (13.8, 15.8)	12.5 (11.9, 13.6)	12 (10.7, 12.9)	−0.6 (−1.93, 0.5)	NS		^***^	^***^	<0.001	12–22
IgG (g/l)	11.2 (9.94, 12.6)	9.97 (8.2, 12)	10.5 (9.01, 12.3)	0.185 (−0.305, 0.88)	NS		^*^		NS	6.7–15
IgA (g/l)	1.72 (1.32, 2.27)	1.93 (1.53, 2.33)	1.9 (1.37, 2.2)	−0.13 (−0.265, 0.0125)	<0.01				NS	0.9–3.7
IgM (g/l)	1.36 (1.15, 1.68)	1.03 (0.76, 1.41)	1.05 (0.68, 1.27)	−0.005 (−0.085, 0.0425)	NS		^***^	^***^	<0.001	0.6–2.2
IgE (IU/ml)	36.6 (12.8, 86.4)	23.9 (9.65, 77.4)	18.4 (8.8, 75.1)	−0.75 (−5.78, 0.325)	NS				NS	0–200

*CRP, C-reactive protein; TAG, triacylglycerols; TSH, thyroid-stimulating hormone; fT4, thyroxine.*

*Changes during hospitalization (calculated as the values at hospital admission and values at hospital discharge) were evaluated by Wilcoxon's paired test corrected for ties. The multiple comparisons between groups were evaluated by the Kruskal-Wallis Z test followed Dunn's multiple comparisons with Bonferonni correction. Both Kruskal-Wallis test and Wilcoxon's test were corrected for multiplicity using Bonferroni correction. The results are shown as median with quartiles. AN1—patients with anorexia nervosa before treatment, AN2—patients with anorexia nervosa after treatment; Δ represents the absolute change calculated as the value after intervention - basal value; nControl = 67, nAN1 = 52, nAN2 = 52;*

* *p < 0.05,*

** *p < 0.01,*

*** *p < 0.001. NS, non-significant*.

### Levels of α-MSH and Anti-α-MSH Antibodies

**Figure 1:**
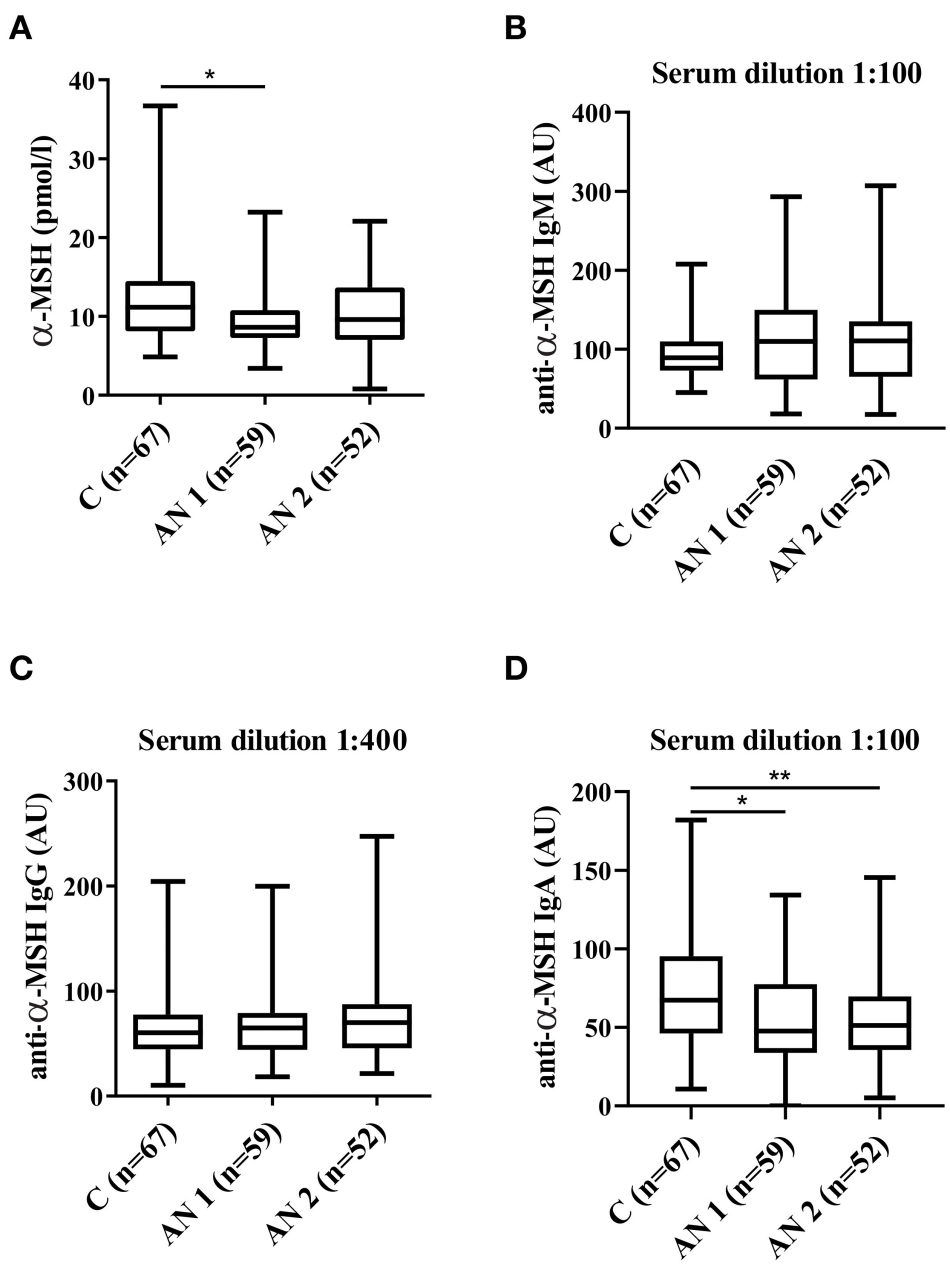
Alpha-melanocyte stimulating hormone (α-MSH) levels. **(A)** α-MSH levels in healthy controls, patients at hospital admission (AN1) and their discharge (AN2). **(B–D)** Levels of anti-α-MSH antibodies (IgM, IgG, and IgA, respectively). ^*^p <0.05, ^**^p <0.01.

Alpha-melanocyte stimulating hormone (α-MSH) levels were determined by commercial radioimmunoassay (RIA) kit (Euro Diagnostica; Figure 1). The minimum detectable α-MSH concentration was 3.0 pmol/L, and the intra- and inter-assay coefficients of variation of the assay were 4.7 and 8.4%, respectively. The cross-reactivity with other proopiomelanocortin peptides (adrenocorticotropic hormone, β-MSH, and γ-MSH) was <0.002%. The radioactivity in the precipitates was measured by Multi Crystal Gamma Counter Berthold LB2111.

Serum levels of autoantibodies against α-MSH were measured by in-house ELISA (Figure 1). Ninety six-well plates (Nunc Immunoplate Maxisorp, Thermo Scientific) were coated with 2 μg/ml of synthetic α-MSH (Bachem AG) in coating buffer (0.5 M Na_2_CO_3_, 0.5 M NaHCO_3_, pH 9.6; 100 μl/well). Plates were incubated for 24 h at 4°C, washed three times with PBS-0.05% Tween, blocked with 1% protease-free BSA in PBS (Sigma-Aldrich), and incubated for 2 h at room temperature. Then, plates were washed (three times with PBS-0.05% Tween) and diluted serum samples (1:50, 1:100, 1:200, 1:400, 1:800, 1:1,600 to determine the optimal serum concentration for each immunoglobulin) in 1% BSA in PBS were loaded and incubated for 2 h at room temperature. After washing (four times with PBS-0.05% Tween), anti-human IgG-HRP (1:4,000 in 1% BSA, Binding Site, UK), anti-human IgA-HRP (1:2,000 in 1% BSA; Binding site), and anti-human IgM-HRP (1:2,000 in 1% BSA; Binding site) were added and incubated for 2 h at room temperature. After the final washing (four times with PBS-0.05% Tween), plates were incubated with substrate solution (3,3′,5,5′-tetramethylbenzidine, TMB; Sigma) for 30 min in darkness at room temperature. The reaction was stopped with 2M H_2_SO_4_. The optical density was determined at 450 and 650 nm by spectrophotometer (Multiskan Ascent Plate Reader, MTX Lab Systems). Blank optical density values (without serum addition) were subtracted. Serum-specific background noise coming from non-coated wells was subtracted from the corresponding OD of the coated wells. Each sample was measured in duplicate. The variation between duplicate values was <5%. The internal standard (reference serum) was used in all plates. The internal standard was prepared by pooling sera variously positive for anti-α-MSH Ab from patients with AN. Serum levels in the standard were assigned as 100 arbitrary units (AU). Sample serum levels were expressed as a percentage of the reference serum optical density in AU.

### Gut Bacterial Composition Analysis

The gut microbiota analysis was performed as described in Prochazkova et al. (24). Briefly, total DNA was isolated from stool samples using the ZymoBIOMICS DNA Miniprep kit (Zymo Research). The primer set with barcodes (342F/806R) was used to amplify the V3-V4 region of the 16S rRNA gene using KAPA HiFi HotStart ReadyMix (Roche). Triplicates of amplicons were pooled, normalized with the SequalPrep^TM^ Normalization Plate Kit (ThermoFisher Scientific), concentrated (3 h, 30°C under vacuum; Concentrator 5301, Eppendorf), purified (DNA Clean & Concentrator Kit, Zymo Research), and ligated with sequencing adapters (TrueSeq DNA PCR-free LT Sample Preparation Kit, Illumina) using the KAPA HyperPlus kit (Roche). The final libraries were pooled in equimolar concentrations and sequenced on an Illumina Miseq using the Miseq reagent Kit v3 (Illumina). The bioinformatic pipeline is described in the Supplementary Materials.

### Correlations Between Anti-α-MSH Antibodies and Bacterial Composition

Using linear models with Gaussian error distribution, we analyzed effect of anti-α-MSH immunoglobulin levels on bacterial Shannon diversity and OTU richness (log transformed). Individual identity was included as random effect and group identity as a covariate. Effect of anti-α-MSH IgA, IgM, IgG levels on microbial betadiversity was analyzed by PERMANOVA, where matrix of among-sample dissimilarities (either Bray-Curtis or Jaccard dissimilarities accounting for OTU relative abundances and prevalences, respectively) was considered as a response and anti-α-MSH IgA, IgM, IgG concentrations as an explanatory variable. Furthermore, we specified individual ids as a ‘strata' (i.e., constrains for permutations), to account for repeated sampling of the same individual. Both alpha and beta analyzes were conducted on rarefied dataset. To test the effect of immunoglobulin levels on abundances of specific OTUs, differential abundance analyses were conducted. Specifically, a vector of read counts for each OTU was entered as a response into Generalized Linear Mixed Model for data with negative binomial distribution, while anti-α-MSH IgA, IgM, IgG concentrations were considered as an explanatory variable, group identity as a covariate and individual identity as a random effect. Furthermore, log-transformed per sample sequencing depth was specified as a model offset (i.e., assuming that read counts for each OTU is proportional to total number of reads per sample). Multiple testing corrections were conducted using FDR method. All anti-α-MSH IgM and anti-α-MSH IgA concentrations exhibited highly skewed distribution. We therefore used their squared-root transformed values (IgA, IgM) or log-transformed values (IgG) in all the statistical analyzes.

### Pro-inflammatory Cytokine Levels

Serum levels of pro-inflammatory cytokines IL-6, IL-17, and TNF-α were determined in duplicate by ELISA (Human IL-6, IL-17, and TNF-α Quantikine HS ELISA kit, Bio-Techne R&D Systems). The minimum detectable concentrations were as follows: TNF-α 0.66 pg/ml; IL-6 0.61 pg/ml; IL-17 0.06 pg/ml. Values were analyzed by one-way Kruskal-Wallis with Dunn's multiple comparisons test. Additionally, multiple correlations of cytokine levels were performed using GraphPad Prism.

## Results

### Anthropometric Measurements

In patients with AN, all weight-related parameters (BMI, body fat, waist and hip circumference) were significantly decreased compared to healthy controls (Table 1). These parameters were lower in patients with AN both at the beginning (AN1) and at the end (AN2) of hospitalization. Although the observed parameter values were significantly lower at hospital discharge compared to healthy controls, all the median values increased during hospitalization (BMI from 14.4 to 17.1 kg/m^2^, body fat from 3 to 9%, waist circumference from 57.5 to 64 cm, and hip circumference from 77 to 83 cm).

### Outcome

We evaluated the treatment outcome of patients with AN at their discharge as the global clinical impression. Eight of 52 patients did not evidence the positive outcome of the therapy, whereas slight or significant improvement was observed in 44 patients with AN. Positive outcome was strongly associated with some anthropometric parameters, such as increase in body fat percentage, hipline and waistline circumference. In addition, it was associated with longer hospitalization and shorter disease duration (Supplementary Table 1). We did not detect any association between positive outcome and biochemical and immunological serum parameters. In our cohort of patients with AN, we did not detect the negative outcome, however the outcome was not evaluated in seven patients with AN that terminated the therapy prematurely.

### Biochemical and Immunological Serum Parameters

The total protein was significantly lower in both patients with AN at hospital admission and their discharge compared to healthy controls. Moreover, we observed a similar trend in AN1 globulin levels (alpha 1-, beta-, and gamma-). At patient discharge, alpha 2- and beta globulin levels increased to the control levels. Conversely, albumin representation percentage was slightly increased in AN1 patients, however, its concentration (g/l) was similar in AN1, AN2, and healthy controls (Table 2).

Serum liver enzyme cholinesterase (CHE) levels were significantly lower in AN1 compared to healthy controls. During hospitalization, CHE levels increased (Table 2). Levels of free thyroxine (fT4), a thyroid gland hormone, were significantly decreased in patients with AN compared to healthy controls. During hospitalization, fT4 levels decreased even more (Table 2).

Further, IgG and IgM antibody levels were decreased in AN1 patients. Whereas IgG isotype levels reached the control values during hospitalization, IgM levels remained the same till therapy end (Table 2). Despite the significant differences between healthy women and patients with AN in the above-mentioned parameters, all the detected protein concentrations were within the normal range (Table 2). In addition to the values shown in Table 2, other serum parameters determining AN1 patients physiological and biochemical states were assessed, and these values were within the normal reference ranges (Supplementary Table 2).

### α-MSH Levels and Antibodies Against α-MSH

The levels of appetite-regulating anorexigenic alpha-melanocyte stimulating hormone (α-MSH) peptide were significantly lower in AN1 patients (Figure 1A). Further, we detected α-MSH autoantibodies in serum of healthy controls and patients with AN. We observed increasing tendency in anti-α-MSH IgM levels in patients with AN (Figure 1B). Anti-α-MSH IgG isotype did not differ between the studied groups (Figure 1C). The levels of anti-α-MSH IgA antibodies were significantly decreased in AN1 and even more so in AN2 patients (Figure 1D).

### Gut Bacterial Composition of Patients With AN and Healthy Controls

**Figure 2:**
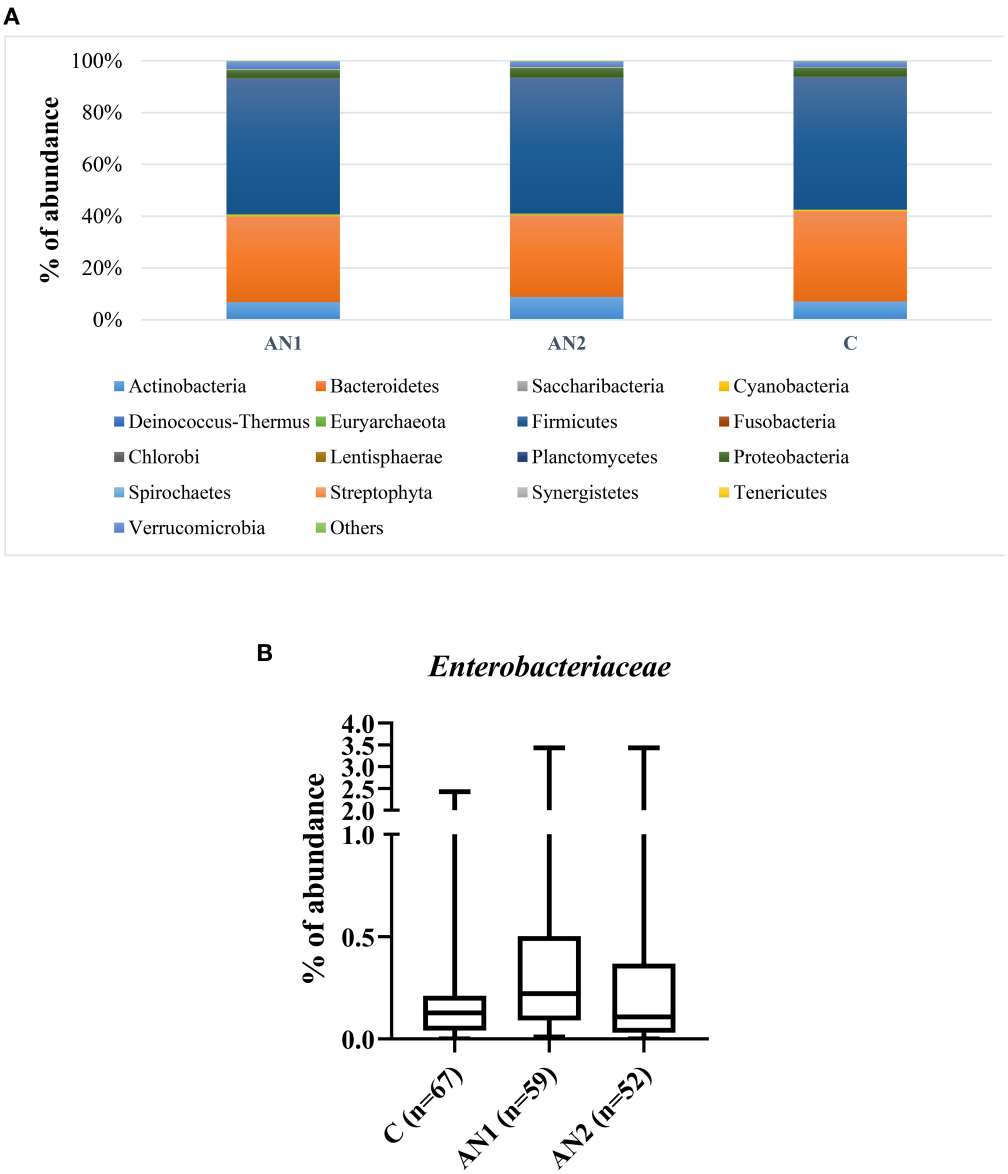
The bacterial phylum composition of healthy controls and patients' gut microbiome **(A)**. The *Enterobacteriaceae* family abundance within these groups **(B)**.

The gut microbiota composition of patients with AN and healthy controls was thoroughly described by our group (24). Here, we show the overall representation of bacterial phylum that does not significantly differ between controls and patients with AN (Figure 2A). More importantly, *Enterobacteriaceae* family abundance tended to increase in AN1 patients (Figure 2B). The average abundance of this family was ~0.33% in healthy controls, 0.53% in AN1, and 0.43% in AN2 patients. However, this difference between the studied groups was not significant.

### Correlations Between Anti-α-MSH Antibodies and Bacterial Composition

**Figure 3:**
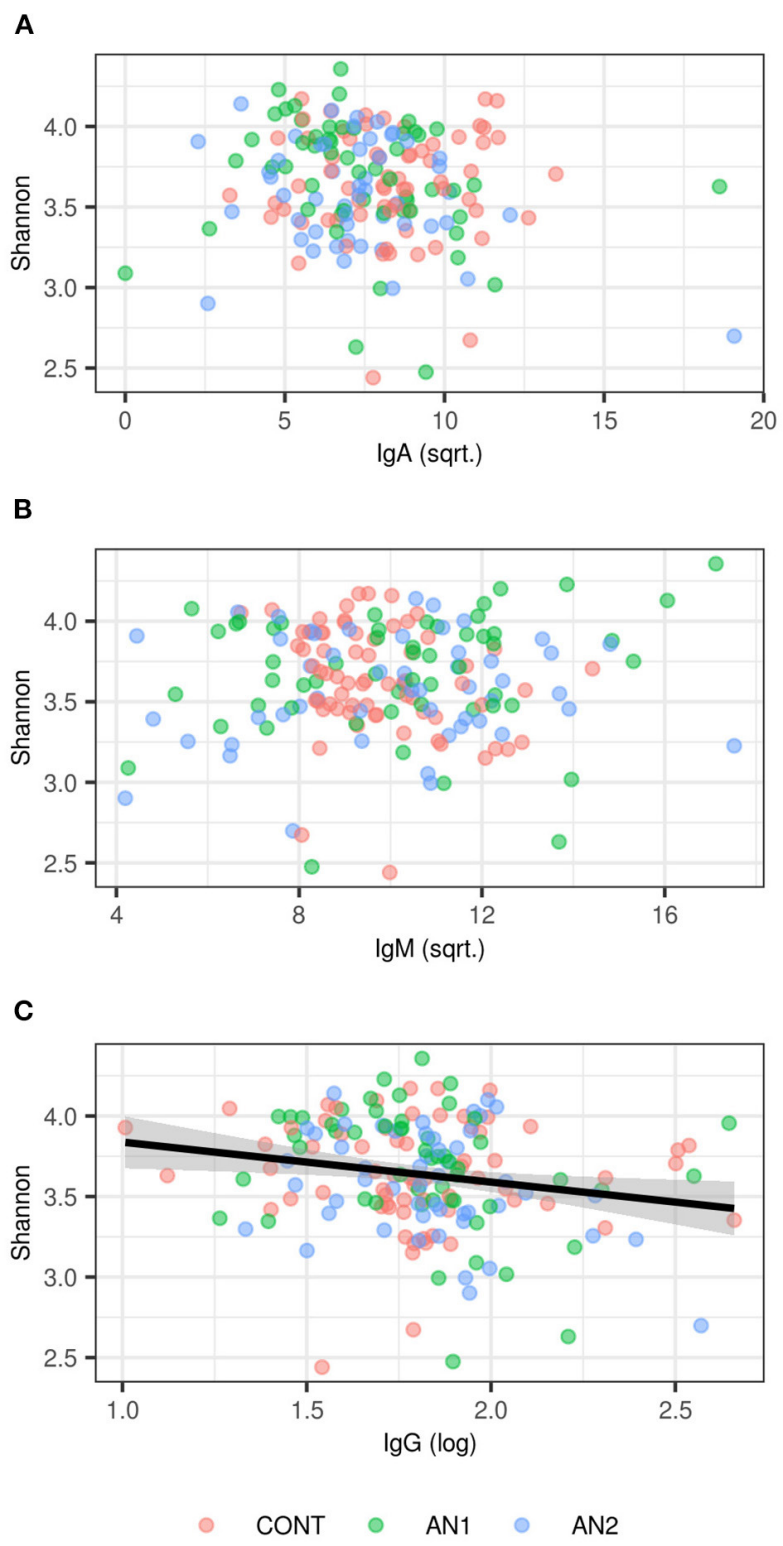
Association between Shannon diversity and **(A)** anti-α-MSH IgA, **(B)** anti-α-MSH IgM, **(C)** anti-α-MSH IgG levels. LMM-based estimates and 95% confidence intervals are shown for significant association (*p* < 0.05).

We did not observe any effect of anti-α-MSH antibodies on OTU richness (*p* > 0.1 in all classes), but anti-α-MSH IgG levels decreased with increasing Shannon diversity index [estimate (±S.E.) = −0.2415 (± 0.0961), *t* = −2.5116, *p* = 0.0129; Figure 3]. However, there was no association of Shannon diversity with anti-α-MSH IgM [estimate = 0.0055 (± 0.0115), *t* = 0.4796, *p* = 0.6322]. The association with anti-α-MSH IgA was only marginally significant [estimate = −0.0207 (± 0.0107), *t* = −1.9222, *p* = 0.0563] and completely disappeared after two outlying samples elimination [estimate = −0.0126 (± 0.0124), *t* = −1.0157, *p* = 0.3110].

PERMANOVA analysis did not find any association between anti-α-MSH antibodies and community composition (Supplementary Table 3).

### Pro-inflammatory Cytokines

**Figure 4:**
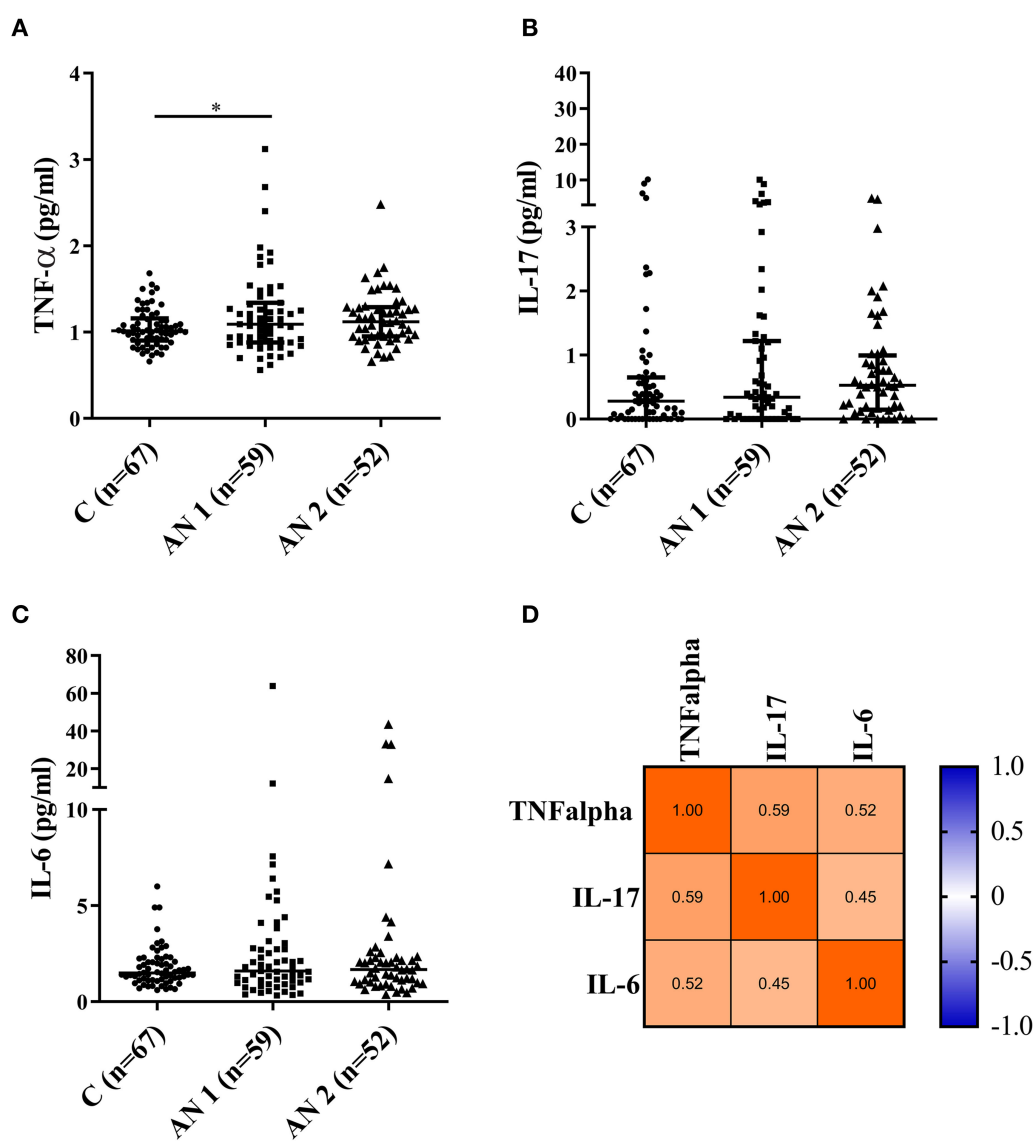
Pro-inflammatory cytokine levels measured in healthy controls, patients at hospital admission (AN1), and their discharge (AN2). **(A–C)** Cytokine levels (TNF-α, IL-17, and IL-6, respectively). Kruskal-Wallis test with Dunn's multiple comparisons test were applied (^*^P < 0.05). The multiple correlations predicted the variables dependency (TNF-α, IL-17, and IL-6; **D**).

We measured the levels of three pro-inflammatory cytokines—TNF-α, IL-17, and IL-6 (Figure 4). The levels were increased in AN1 and AN2 compared to controls, however only the increase in AN1 TNF-α levels was significant (Figure 4A). Multiple correlation analyses of TNF-α, IL-17, and IL-6 levels in AN1 predicted dependency of variables, suggesting that some patients exerted increased immune reactivity (Figure 4D).

## Discussion

We recruited 45 restrictive and 14 purgative patients suffering from anorexia nervosa (AN). The aim was to evaluate the effect of realimentation on classical biochemical parameters and specific parameters, especially those reflecting the state of the AN patient's immune system. Patients with AN exerted significantly decreased anthropometric parameter values connected with their reduced food intake—body mass index (BMI), body fat, and waist and hip circumferences (Table 1). All these parameters improved during hospitalization, although they did not reach the healthy control values. We did not detect significant differences in any of the observed parameters between restrictive and purgative AN inpatients. Interestingly, a greater proportion of purgative patients with AN terminated the therapy prematurely (4 out of 14 purgative AN compared to 3 out of 45 restrictive AN). Therefore, reliable statistical analysis could not be performed with the obtained data. Outcome represents the global impression at the patient's discharge. It includes patient's attitude to weight gain, the motivation to maintain this weight gain, the perception of their body, BMI gain, and risk factors for possible relapse. Most of the patients that did not terminate the therapy prematurely, reached the positive treatment outcome (Supplementary Table 1).

In AN patient serum, total protein levels were decreased as compared to healthy controls. At hospital discharge, the levels increased slightly. Umeki also showed an abnormal total serum protein decrease in 93% of patients with AN recruited to the study, with its significant improvement at discharge (25). In another study, the total protein level was significantly lower in patients suffering from AN more than 5 years compared to those with short-term illness duration (26). The albumin concentrations were similar in all three studied groups; however, the albumin percentage was increased in AN1 patients. Related to this finding, alpha 1-, beta-, and gamma globulins representation were decreased in AN1 patients. During hospitalization, alpha 2- and beta globulin levels increased (Table 2). Similar biochemical parameters were previously shown in patients with severe and enduring AN (27). The observed data support the fact that the energy metabolism is altered in patients with AN (28).

Cholinesterase (CHE) is an enzyme considered as an overall hepatocyte function marker and therefore can be influenced by nutritional status. Mice loose about 40% of their liver protein content within 48 h of starvation (29). We detected decreased CHE levels in AN1 patients compared to healthy controls (Table 2). Previously, Montagnese et al. showed abnormalities in serum liver enzymes in underweight patients suffering from eating disorders (30). They suggested CHE as a marker of the effect of primary malnutrition on liver function (30), which is in accordance with our results.

AN is associated with endocrine dysregulation, which is likely an adaptive state of long-term food restriction (31). Abnormalities in thyroid function connected with starvation, protein-energy undernutrition, and anorexia nervosa were described previously (32). We detected significantly reduced free thyroxine (fT4) levels in AN1 patients, and even lower in AN2 (Table 2). This suggests that the reduced fT4 levels are independent of the patients' nutritional status. The persistence of the endocrine changes after the hospitalization was suggested to contribute to susceptibility to AN recurrence (33). Similarly, lower total and free fT4 levels compared to controls were described in patients with AN (34). We did not find a significant change in thyroid-stimulating hormone levels (TSH, Table 2), a pituitary glycoprotein that regulates the thyroid gland to produce thyroxine. The normal TSH level in the presence of the low plasma fT3 and fT4 concentrations is called euthyroid sick syndrome. This non-thyroid disorder can be observed besides others in patients with AN (35).

Patients with AN are not able to adapt their eating behavior to energy requirements, which can be influenced by their appetite-regulating hormones dysregulation (32). We observed decreased levels of anorexigenic peptide alpha-melanocyte stimulating hormone (α-MSH) produced by the anterior pituitary in AN1 patients (Figure 1A). Plasma α-MSH levels were lower in both patients with AN and constitutionally thin (CT) women, nevertheless the levels significantly increased during lunchtime in CT individuals as compared to AN women (12). The majority of patients with AN were described to display autoantibodies reacting with α-MSH (36). Interestingly, Fetissov et al. described significantly increased levels of IgM anti-α-MSH antibodies in patients' serum (16). This is in accordance with our results because elevated levels of anti-α-MSH IgM antibodies were detected in AN1 and AN2, although the increase was not significant (Figure 1B). This finding could be explained by the differences in ELISA assays evaluation. Our group used the arbitrary units based on the internal standard for antibody levels determination, while the Fetissov group measured the optical density. Besides, total IgM levels were decreased in AN1 and AN2 patients (Table 2), suggesting the greater relative share of these anti-α-MSH autoantibodies. Levels of IgG and IgM autoantibodies against α-MSH correlated with the Eating Disorder Inventory score in AN and bulimia nervosa patients, respectively (16). We detected significantly decreased levels of anti-α-MSH IgA in AN1 and AN2 (Figure 1D). To our knowledge anti-α-MSH IgA levels have not been determined in patients with AN before. IgA antibodies are primarily products of mucosal cellular immune response. Patients with AN often suffer from various GIT disorders, and thus the mucosal immunity and IgA production may be affected (37, 38). However, total serum IgA levels were determined to be similar in patients with AN and healthy controls. Recently Fitzpatrick and colleagues showed the presence of IgA-secreting plasma cells educated in the gut positioned adjacent to dural venous sinuses in human and mouse brain. These cells were almost completely absent in germ-free mice and substantially reduced after 6 weeks of mice oral antibiotics treatment (39). These data suggest that the presence of meningeal IgA-secreting cells is dependent on the gut microbiome. The production of IgA directed against various appetite-regulating peptides will be further studied.

Caseinolytic protease B (ClpB), bacterial heat shock chaperone protein produced by some *Enterobacteriaceae* family representatives, induces the production of antibodies cross-reacting with α-MSH (17). Importantly, bioinformatics analysis showed that ClpB from the *Enterobacteriaceae* family displayed the α-MSH-like functional motifs (40). To assess the connection between anti-α-MSH antibodies and *Enterobacteriaceae*, we determined the family abundance in the gut microbiome of patients with AN and healthy controls (Figure 2). Our group described that the gut microbiota was very similar at the phylum level, although microbiota depletion signs were observed in patients with AN (24). *Enterobacteriaceae* abundance was increased in patients with AN, but this increase was not significant, most likely due to great interindividual variability. Supposing that not all representatives of this family produce ClpB inducing α-MSH-cross-reactive antibody production, the focus on specific bacterial species could bring more information. This approach would require deeper sequencing analysis to detect individual *Enterobacteriaceae* family species. Moreover, with the increasing number of bacterial sequences deposited in databases, the exclusive role of *Enterobacteriaceae* in anti-α-MSH antibody production is questionable because other bacteria express protein with potential α-MSH-like motif (41). This has to be taken into account in further analyses. In addition, we observed the association between decreasing levels of anti-α-MSH IgG and increasing Shannon diversity index (Figure 3). These data suggest that increased diversity of gut bacterial species may result in decreased abundance of species expressing ClpB protein with α-MSH-like motif, which triggers production of α-MSH cross-reactive autoantibodies. With the increasing knowledge about bidirectional communication on the microbiome-gut-brain axis, its effect on the development of various neuropsychiatry disorders is more and more apparent (42, 43).

Nutrition deprivation, especially protein-energy malnutrition, leads to increased infection frequency and severity. This is caused by various secondary immunodeficiency types including cell-mediated immunity and humoral response (44). However, the immune impairments are less frequent and less severe in individuals suffering from eating disorders considering their highly defective nutritional status. Nevertheless, immune system dysregulation was found at both the native and adaptive immunity levels (20). Although deficits in cellular immunity were frequently detected (45, 46), studies describing adaptive humoral immunity disturbances are inconclusive. One study described normal serum immunoglobulin levels in patients with AN (47), while an other showed elevated IgG and IgM levels in a small group of patients with AN compared to healthy controls (48). IgA and IgE serum levels were similar to those of healthy controls (Table 2). IgG levels were significantly lower upon admission and increased to the control level during hospitalization. IgM levels were decreased both at therapy beginning and end (Table 2). Although IgG and IgM levels were significantly altered from controls, their values were within normal reference ranges (Table 2). However, it is important to realize that these reference values are based on the values of normal weight healthy individuals.

Regarding the cytokine profile in patients with AN, results from different studies vary. Generally, upregulated levels of pro-inflammatory cytokines were usually detected (49, 50). These cytokines are responsible for the local inflammatory response and synchronize the physiological and behavioral components of a systemic infection response. Their increased production upon infection can result in the development of so-called sickness behavior characterized by appetite loss, reduction in activity and social interactions, and depressed mood (51). We observed elevated levels of TNF-α, IL-17, and IL-6 in patients with AN, however, only TNF-α levels increased significantly in patients with AN at hospital admission (Figure 4). The multiple correlation predicted the dependency of individual cytokines, showing that some patients with AN exerted an increased inflammatory response (Figure 4D). Via their effect on appetite reduction, the cytokines may participate in the promotion of AN from the early disease stage to the chronic severe and enduring AN. This possible association will be further studied.

To conclude, we detected significant differences in basal biochemical parameters in patients with AN compared to healthy controls, showing the negative effect of malnutrition on various organs such as the liver and thyroid gland. Importantly, we found the alterations in immune system parameters—elevated levels of pro-inflammatory cytokines, decreased anti-α-MSH IgA levels compared to healthy controls and the association between anti-α-MSH IgG levels and gut bacterial diversity.

## Data Availability Statement

The raw data supporting the conclusions of this article will be made available by the authors, without undue reservation.

## Ethics Statement

The studies involving human participants were reviewed and approved by Ethics Committee of General University Hospital in Prague. The patients/participants provided their written informed consent to participate in this study. The number of approved protocol (16/16; approval date 21/4/2016).

## Author Contributions

RR prepared the manuscript. All authors contributed to the conception and design of the study as well as data collection, analysis, result interpretation, revised, and approved the final version.

## Conflict of Interest

The authors declare that the research was conducted in the absence of any commercial or financial relationships that could be construed as a potential conflict of interest.

## Publisher's Note

All claims expressed in this article are solely those of the authors and do not necessarily represent those of their affiliated organizations, or those of the publisher, the editors and the reviewers. Any product that may be evaluated in this article, or claim that may be made by its manufacturer, is not guaranteed or endorsed by the publisher.

## References

[1] Zipfel S Giel KE Bulik CM Hay P Schmidt U. Anorexia nervosa: aetiology, assessment, and treatment. Lancet Psychiatry. (2015) 2:1099–111. 10.1016/S2215-0366(15)00356-9 26514083

[2] Arcelus J Mitchell AJ Wales J Nielsen S. Mortality rates in patients with anorexia nervosa and other eating disorders. A meta-analysis of 36 studies. Arch Gen Psychiatry. (2011) 68:724–31. 10.1001/archgenpsychiatry.2011.74 21727255

[3] Borgo F Riva A Benetti A Casiraghi MC Bertelli S Garbossa S. Microbiota in anorexia nervosa: the triangle between bacterial species, metabolites and psychological tests. PLoS ONE. (2017) 12:e0179739. 10.1371/journal.pone.0179739 28636668 PMC5479564

[4] Kleiman SC Watson HJ Bulik-Sullivan EC Huh EY Tarantino LM Bulik CM. The Intestinal Microbiota in Acute Anorexia Nervosa and During Renourishment: Relationship to Depression, Anxiety, and Eating Disorder Psychopathology. Psychosom Med. (2015) 77:969–81. 10.1097/PSY.0000000000000247 26428446 PMC4643361

[5] Morita C Tsuji H Hata T Gondo M Takakura S Kawai K. Gut dysbiosis in patients with anorexia nervosa. PLoS ONE. (2015) 10:e0145274. 10.1371/journal.pone.0145274 26682545 PMC4687631

[6] Rosenbaum M Knight R Leibel RL. The gut microbiota in human energy homeostasis and obesity. Trends Endocrinol Metab. (2015) 26:493–501. 10.1016/j.tem.2015.07.002 26257300 PMC4862197

[7] Benini L Todesco T Dalle Grave R Deiorio F Salandini L Vantini I. Gastric emptying in patients with restricting and binge/purging subtypes of anorexia nervosa. Am J Gastroenterol. (2004) 99:1448–54. 10.1111/j.1572-0241.2004.30246.x 15307858

[8] Prochazkova P Roubalova R Dvorak J Tlaskalova-Hogenova H Cermakova M Tomasova P. Microbiota, microbial metabolites, and barrier function in a patient with anorexia nervosa after fecal microbiota transplantation. Microorganisms. (2019) 7:338. 10.3390/microorganisms7090338 31510101 PMC6780752

[9] Westmoreland P Krantz MJ Mehler PS. Medical Complications of Anorexia Nervosa and Bulimia. Am J Med. (2016) 129:30–7. 10.1016/j.amjmed.2015.06.031 26169883

[10] Baskaran C Misra M Klibanski A. Effects of anorexia nervosa on the endocrine system. Pediatr Endocrinol Rev. (2017) 14:302–11. 10.17458/per.vol14.2017.BMK.effectsanorexianervosa 28508601

[11] Monteleone P Maj M. Dysfunctions of leptin, ghrelin, BDNF and endocannabinoids in eating disorders: beyond the homeostatic control of food intake. Psychoneuroendocrinology. (2013) 38:312–30. 10.1016/j.psyneuen.2012.10.021 23313276

[12] Galusca B Prevost G Germain N Dubuc I Ling Y Anouar Y. Neuropeptide Y and alpha-MSH circadian levels in two populations with low body weight: anorexia nervosa and constitutional thinness. PLoS ONE. (2015) 10:e0122040. 10.1371/journal.pone.0122040 25798605 PMC4370702

[13] Schalla MA Stengel A. The role of ghrelin in anorexia nervosa. Int J Mol Sci. (2018) 19:2117. 10.3390/ijms19072117 PMC6073411 30037011

[14] Takagi K Legrand R Asakawa A Amitani H Francois M Tennoune N. Anti-ghrelin immunoglobulins modulate ghrelin stability and its orexigenic effect in obese mice and humans. Nat Commun. (2013) 4:2685. 10.1038/ncomms3685 24158035 PMC3826639

[15] Vaeroy H Adori C Legrand R Lucas N Breton J Cottard C. Autoantibodies reactive to adrenocorticotropic hormone can alter cortisol secretion in both aggressive and nonaggressive humans. Proc Natl Acad Sci USA. (2018) 115:E6576–584. 10.1073/pnas.1720008115 29941562 PMC6048475

[16] Fetissov SO Harro J Jaanisk M Jarv A Podar I Allik J. Autoantibodies against neuropeptides are associated with psychological traits in eating disorders. Proc Natl Acad Sci USA. (2005) 102:14865–70. 10.1073/pnas.0507204102 16195379 PMC1253594

[17] Tennoune N Chan P Breton J Legrand R Chabane YN Akkermann K. Bacterial ClpB heat-shock protein, an antigen-mimetic of the anorexigenic peptide alpha-MSH, at the origin of eating disorders. Transl Psychiatry. (2014) 4:e458. 10.1038/tp.2014.98 25290265 PMC4350527

[18] Lucas N Legrand R Bole-Feysot C Breton J Coeffier M Akkermann K. Immunoglobulin G modulation of the melanocortin 4 receptor signaling in obesity and eating disorders. Transl Psychiatry. (2019) 9:87. 10.1038/s41398-019-0422-9 30755592 PMC6372612

[19] Tylee DS Sun J Hess JL Tahir MA Sharma E Malik R. Genetic correlations among psychiatric and immune-related phenotypes based on genome-wide association data. Am J Med Genet B Neuropsychiatr Genet. (2018) 177:641–57. 10.1002/ajmg.b.32652 30325587 PMC6230304

[20] Gibson D Mehler PS. Anorexia nervosa and the immune system-a narrative review. J Clin Med. (2019) 8:1915. 10.3390/jcm8111915 31717370 PMC6912362

[21] Nova E Marcos A. Nutritional status and immunocompetence in eating disorders. In: Strumia R, editor. Eating Disorders and the Skin. Berlin Heidelberg: Springer (2012). p. 37–45.

[22] American Psychiatric Association. Diagnostic and Statistical Manual of Mental Disorders. 5th ed. Arlington, VA (2013).

[23] Garner DM. Eating Disorder Inventory-2 Professional Manual. Odessa, FL: Psychological Assessment Resources (1991).

[24] Prochazkova P Roubalova R Dvorak J Kreisinger J Hill M Tlaskalova-Hogenova H. The intestinal microbiota and metabolites in patients with anorexia nervosa. Gut Microbes. (2021) 13:1–25. 10.1080/19490976.2021.1902771 33779487 PMC8018350

[25] Umeki S. Biochemical abnormalities of the serum in anorexia nervosa. J Nerv Ment Dis. (1988) 176:503–6. 10.1097/00005053-198808000-00009 2457069

[26] Takakura S Aso CS Toda K Hata T Yamashita M Sudo N. Physical and psychological aspects of anorexia nervosa based on duration of illness: a cross-sectional study. Biopsychosoc Med. (2019) 13:32. 10.1186/s13030-019-0173-0 31889996 PMC6929428

[27] Narayanan V Gaudiani JL Mehler PS. Serum albumin levels may not correlate with weight status in severe anorexia nervosa. Eat Disord. (2009) 17:322–6. 10.1080/10640260902991202 19548148

[28] Winston AP. The clinical biochemistry of anorexia nervosa. Ann Clin Biochem. (2012) 49:132–43. 10.1258/acb.2011.011185 22349551

[29] Mortimore GE Hutson NJ Surmacz CA. Quantitative correlation between proteolysis and macro- and microautophagy in mouse hepatocytes during starvation and refeeding. Proc Natl Acad Sci USA. (1983) 80:2179–83. 10.1073/pnas.80.8.2179 6340116 PMC393781

[30] Montagnese C Scalfi L Signorini A De Filippo E Pasanisi F Contaldo F. Cholinesterase and other serum liver enzymes in underweight outpatients with eating disorders. Int J Eat Disord. (2007) 40:746–50. 10.1002/eat.20432 17610252

[31] Schorr M Miller KK. The endocrine manifestations of anorexia nervosa: mechanisms and management. Nat Rev Endocrinol. (2017) 13:174–86. 10.1038/nrendo.2016.175 27811940 PMC5998335

[32] Estour B Germain N Diconne E Frere D Cottet-Emard JM Carrot Hormonal profile heterogeneity and short-term physical risk in restrictive anorexia nervosa. J Clin Endocrinol Metab. (2010) 95:2203–10. 10.1210/jc.2009-2608 20305007

[33] Lawson EA Klibanski A. Endocrine abnormalities in anorexia nervosa. Nat Clin Pract Endocrinol Metab. (2008) 4:407–14. 10.1038/ncpendmet0872 18542109

[34] Misra M Miller KK Almazan C Ramaswamy K Lapcharoensap W Worley M. Alterations in cortisol secretory dynamics in adolescent girls with anorexia nervosa and effects on bone metabolism. J Clin Endocrinol Metab. (2004) 89:4972–80. 10.1210/jc.2004-0723 15472193

[35] Van den Berghe G. Non-thyroidal illness in the ICU: a syndrome with different faces. Thyroid. (2014) 24:1456–65. 10.1089/thy.2014.0201 24845024 PMC4195234

[36] Fetissov SO Hallman J Oreland L Af Klinteberg B Grenback E Hulting AL. Autoantibodies against alpha -MSH, ACTH, and LHRH in anorexia and bulimia nervosa patients. Proc Natl Acad Sci USA. (2002) 99:17155–60. 10.1073/pnas.222658699 12486250 PMC139285

[37] Gabriel T Paul S Berger A Massoubre C. Anorexia nervosa and autism spectrum disorders: future hopes linked to mucosal immunity. Neuroimmunomodulation. (2019) 26:265–75. 10.1159/000502997 31715599

[38] Schenk M Mueller C. The mucosal immune system at the gastrointestinal barrier. Best Pract Res Clin Gastroenterol. (2008) 22:391–409. 10.1016/j.bpg.2007.11.002 18492562

[39] Fitzpatrick Z Frazer G Ferro A Clare S Bouladoux N Ferdinand J. Gut-educated IgA plasma cells defend the meningeal venous sinuses. Nature. (2020) 587:472–76. 10.1038/s41586-020-2886-4 33149302 PMC7748383

[40] Fetissov SO Legrand R Lucas N. Bacterial protein mimetic of peptide hormone as a new class of protein- based drugs. Curr Med Chem. (2019) 26:546–53. 10.2174/0929867324666171005110620 28982315

[41] Arnoriaga-Rodriguez M Mayneris-Perxachs J Burokas A Perez-Brocal V Moya A Portero-Otin M. Gut bacterial ClpB-like gene function is associated with decreased body weight and a characteristic microbiota profile. Microbiome. (2020) 8:59. 10.1186/s40168-020-00837-6 32354351 PMC7193372

[42] Roubalova R Prochazkova P Papezova H Smitka K Bilej M Tlaskalova-Hogenova H. Anorexia nervosa: gut microbiota-immune-brain interactions. Clin Nutr. (2019) 39:676–84. 10.1016/j.clnu.2019.03.023 30952533

[43] Dinan TG Cryan JF. The microbiome-gut-brain axis in health and disease. Gastroenterol Clin North Am. (2017) 46:77–89. 10.1016/j.gtc.2016.09.007 28164854

[44] Bourke CD Berkley JA Prendergast AJ. Immune dysfunction as a cause and consequence of malnutrition. Trends Immunol. (2016) 37:386–98. 10.1016/j.it.2016.04.003 27237815 PMC4889773

[45] Elegido A Graell M Andres P Gheorghe A Marcos A Nova E. Increased naive CD4(+) and B lymphocyte subsets are associated with body mass loss and drive relative lymphocytosis in anorexia nervosa patients. Nutr Res. (2017) 39:43–50. 10.1016/j.nutres.2017.02.006 28385288

[46] Palmblad J Fohlin L Lundstrom M. Anorexia nervosa and polymorphonuclear (PMN) granulocyte reactions. Scand J Haematol. (1977) 19:334–42. 10.1111/j.1600-0609.1977.tb01483.x 918561

[47] Golla JA Larson LA Anderson CF Lucas AR Wilson WR Tomasi TB Jr.. An immunological assessment of patients with anorexia nervosa. Am J Clin Nutr. (1981) 34:2756–62. 10.1093/ajcn/34.12.2756 6797290

[48] Wyatt RJ Farrell M Berry PL Forristal J Maloney MJ West CD. Reduced alternative complement pathway control protein levels in anorexia nervosa: response to parenteral alimentation. Am J Clin Nutr. (1982) 35:973–80. 10.1093/ajcn/35.5.973 6805290

[49] Dalton B Bartholdy S Robinson L Solmi M Ibrahim MAA Breen G. A meta-analysis of cytokine concentrations in eating disorders. J Psychiatr Res. (2018) 103:252–64. 10.1016/j.jpsychires.2018.06.002 29906710

[50] Solmi M Veronese N Favaro A Santonastaso P Manzato E Sergi G. Inflammatory cytokines and anorexia nervosa: A meta-analysis of cross-sectional and longitudinal studies. Psychoneuroendocrinology. (2015) 51:237–52. 10.1016/j.psyneuen.2014.09.031 25462897

[51] Maier SF Watkins LR. Cytokines for psychologists: implications of bidirectional immune-to-brain communication for understanding behavior, mood, and cognition. Psychol Rev. (1998) 105:83–107. 10.1037/0033-295X.105.1.83 9450372

