# Supplementary material for: Altered Serum Immunological and Biochemical Parameters and Microbiota Composition in Patients With AN During Realimentation
Source: Front Nutr. 2021 Aug 2;8:680870. doi: 10.3389/fnut.2021.680870 (PMC8365021; doi:10.3389/fnut.2021.680870)
Supplement: Supplementary file 1 [file Data_Sheet_1.docx]

Supplementary Material

**Table S1:** Relationship between relevant parameters and outcome of patients with AN after hospitalization: no change (n=8), slight or significant improvement (n=44).

| **Variable** | **Outcome** | | |  |
| --- | --- | --- | --- | --- |
|  | **No change** | **Improvement** | **p-value** |  |
| Hospitalization (days) | 25 (20, 49) | 53.5 (42, 64.3) | 0.022 | * |
| Disease duration (months) | 108 (81, 243) | 54 (18, 120) | 0.020 | * |
| ∆BMI (kg/m^2^) | 1.34 (0.8, 1.95) | 2.35 (1.71, 3.36) | 0.023 | * |
| ∆Body Fat (%) | 0 (0, 0) | 4.4 (1.85, 7.85) | ˂0.001 | *** |
| ∆Waist (cm) | 4 (2.5, 5) | 6 (4.25, 8) | 0.028 | * |
| ∆Hip (cm) | 2 (0.75, 3) | 5 (3, 7) | 0.017 | * |

n-Whitney test with the normal approximation with continuity correction was used. The results are shown as median with quartiles. *p˂0.05, ***p˂0.001

**Table S2:** Initial serum values of patients with AN at hospital admission and the reference ranges. The values represent medians with quartiles.

| **Variable** | **AN1 (medians with quartiles)** | **Normal adult women reference ranges** |
| --- | --- | --- |
| **Bilirubin (umol/l)** | 7.9 (5.8, 13) | 2.0 – 17.0 |
| **ALT (ukat/l)** | 0.425 (0.298, 0.73) | 0.1 - 0.78 |
| **AST (ukat/l)** | 0.405 (0.33, 0.51) | 0.1 - 0.72 |
| **GGT (ukat/l)** | 0.385 (0.288, 0.605) | 0.14 - 0.68 |
| **ALP (ukat/l)** | 0.84 (0.703, 1.04) | 0.66 - 2.2 |
| **AMS (ukat/l)** | 1.41 (1.09, 1.76) | 0.47 - 1.67 |
| **AMS-P (ukat/l)** | 0.57 (0.45, 0.735) | 0.0 - 0.88 |
| **Cholesterol (mmol/l)** | 4.31 (3.86, 5.22) | 2.9 – 5.0 |
| **Urea (mmol/l)** | 4.6 (4, 5.5) | 2.0 - 6.7 |
| **Creatinine (μmol/l)** | 65 (59, 74) | 44.0 – 104.0 |
| **Uric acid (μmol/l)** | 199 (167, 227) | 140.0 – 340.0 |
| **Na (mmol/l)** | 141 (139, 142) | 137.0 – 146.0 |
| **K (mmol/l)** | 4.4 (4.1, 4.6) | 3.8 – 5.0 |
| **Cl (mmol/l)** | 101 (100, 103) | 97.0 – 108.0 |
| **Ca (mmol/l)** | 2.34 (2.28, 2.38) | 2.0 - 2.75 |
| **P (mmol/l)** | 1.19 (1.02, 1.31) | 0.65 - 1.61 |
| **Mg (mmol/l)** | 0.83 (0.8, 0.875) | 0.7 – 1.0 |

ALT – alanine transaminase, AST – aspartate transaminase, GGT - γ-glutamyltransferase, ALP – alkaline phosphatase, AMS – total serum amylase, AMS-P – pancreatic amylase

***Bioinformatic pipeline***

Fastq files produced by Illumina Miseq were demultiplexed and primers were trimmed by *skewer* software [1].  Using *dada2* [2] we eliminated low-quality sequences (expected number of errors per read > 1), denoised quality-filtered fastq files, and constructed an abundance matrix (OTU table) representing read counts for individual haplotypes in each sample. Next, we identified chimeric haplotypes using *uchime* [3] and the gold.fna database (bacterial data) or UNITE database [4] (fungal data) and eliminated them from the OTU table. Using Procrustean analyses, we checked for consistency in haplotype composition among profiles of identical samples that differed only in the sequencing orientation (i.e. 3' to 5' end or 5' to 3' end), and retained only those haplotypes that were consistently present in both duplicates. After these steps, haplotypes were clustered to Operational Taxonomic Units (OTUs) using *vsearch* [5], assuming 97% sequence similarity threshold. Taxonomic assignation of OTUs was conducted by *RDP classifier* (80% confidence threshold) [6]  and Silva reference database (v. 132) [7], (bacterial data) or UNITE database (fungal data) [4]. In specific cases, we applied phylogenetic placement analyses to achieve more detailed OTU assignation. To do so, we extracted all reference 16S rRNA sequences corresponding to the same genus as the OTUs in question from the Silva database, and clustered them at 99% similarity using *vsearch* [5]. Representative sequences for clusters exhibiting >97% sequence similarity with any OTU in question were used for phylogenetic reconstruction, which was done by *RAxML* [8], assuming the GTRI substitution model after *mafft* alignment [9]. Bootstrap analysis (1000 replicates) was conducted to assess phylogenetic clade robustness. OTU table, OTU representative sequences, OTU taxonomy, and sample metadata were merged into a single phyloseq database for later statistical calculations [10].

**Table S3:** Effect of anti-α-MSH antibodies levels on bacterial composition analyzed by PERMANOVA. Degrees of freedom (Df), mean sum of squares, value of (pseudo-) F statistics (F), proportion of explained variation (R^2^), and probability values (p) are shown. Bray-Curtis or Jaccard dissimilarities were used as betadiversity indices.

|  |  | **Bray-Curtis** | | | | **Jaccard** | | | |
| --- | --- | --- | --- | --- | --- | --- | --- | --- | --- |
|  | **Df** | **MeanSqs** | **F** | **R^2^** | **p** | **MeanSqs** | **F** | **R^2^** | **p** |
| **anti-α-MSH IgA** | 1  167 | 0.3060  0.2246 | 1.3624 | 0.0079  0.9658 | 0.2050 | 0.2186  0.1431 | 1.5271 | 0.0087  0.9566 | 0.1250 |
| **anti-α-MSH IgM** | 1  167 | 0.2560  0.2249 | 1.1382 | 0.0066  0.9671 | 0.3700 | 0.1562  0.1435 | 1.0886 | 0.0063  0.9591 | 0.4900 |
| **anti-α-MSH IgG** | 1  167 | 0.3410  0.2244 | 1.5200 | 0.0088  0.9649 | 0.3900 | 0.2023  0.1432 | 1.4125 | 0.0081  0.9572 | 0.5800 |

1. Jiang, H., et al., *Skewer: a fast and accurate adapter trimmer for next-generation sequencing paired-end reads.* BMC Bioinformatics, 2014. **15**: p. 182.

2. Callahan, B.J., et al., *DADA2: High-resolution sample inference from Illumina amplicon data.* Nat Methods, 2016. **13**(7): p. 581-3.

3. Edgar, R.C., et al., *UCHIME improves sensitivity and speed of chimera detection.* Bioinformatics, 2011. **27**(16): p. 2194-200.

4. Nilsson, R.H., et al., *The UNITE database for molecular identification of fungi: handling dark taxa and parallel taxonomic classifications.* Nucleic Acids Res, 2019. **47**(D1): p. D259-D264.

5. Rognes, T., et al., *VSEARCH: a versatile open source tool for metagenomics.* PeerJ, 2016. **4**: p. e2584.

6. Wang, Q., et al., *Naive Bayesian classifier for rapid assignment of rRNA sequences into the new bacterial taxonomy.* Appl Environ Microbiol, 2007. **73**(16): p. 5261-7.

7. Quast, C., et al., *The SILVA ribosomal RNA gene database project: improved data processing and web-based tools.* Nucleic Acids Res, 2013. **41**: p. D590-6.

8. Stamatakis, A., *RAxML version 8: a tool for phylogenetic analysis and post-analysis of large phylogenies.* Bioinformatics, 2014. **30**(9): p. 1312-3.

9. Katoh, K. and D.M. Standley, *MAFFT multiple sequence alignment software version 7: improvements in performance and usability.* Mol Biol Evol, 2013. **30**(4): p. 772-80.

10. McMurdie, P.J. and S. Holmes, *phyloseq: an R package for reproducible interactive analysis and graphics of microbiome census data.* PLoS One, 2013. **8**(4): p. e61217.
